# Operant novelty seeking predicts cue-induced reinstatement following cocaine but not water reinforcement in male rats

**DOI:** 10.1007/s00213-023-06441-4

**Published:** 2023-08-08

**Authors:** Amy M. Gancarz, Devin P. Hagarty, Moriah M. Cobb, Michael A. Kausch, Brandon Krieg, Nora Alammari, Kameron Gilbert, Jacqueline Russo, David M. Dietz

**Affiliations:** 1 Department of Psychology, California State University, Bakersfield, Dorothy Donahoe Hall (DDH) H106, 9001 Stockdale Highway, Bakersfield, CA 93311 USA; 2 Clinical and Research Institute On Addictions, University at Buffalo, Buffalo, NY USA; 3 Department of Pharmacology and Toxicology, Jacobs School of Medicine and Biomedical Sciences, University at Buffalo, Buffalo, NY USA

**Keywords:** Cocaine, Drug abuse, Self-administration, Operant conditioning, Rat, Sensation seeking, Novelty

## Abstract

**Rationale:**

An important facet of cocaine addiction is a high propensity to relapse, with increasing research investigating factors that predispose individuals toward uncontrolled drug use and relapse. A personality trait linked to drug addiction is high sensation seeking, i.e., a preference for novel sensations/experiences. In an animal model of sensation seeking, operant novelty seeking predicts the acquisition of drug self-administration.

**Objective:**

The primary goal of this research was to evaluate the hypothesis that sensitivity to the reinforcing effects of novel sensory stimuli predicts more intensive aspects of drug-taking behaviors, such as relapse.

**Methods:**

Rats were first tested for Operant Novelty Seeking, during which responses resulted in complex visual/auditory stimuli. Next, rats were trained to respond to water/cocaine reinforcers signaled by a cue light. Finally, rats were exposed to extinction in the absence of discrete cues and subsequently tested in a single session of cue-induced reinstatement, during which active responses resulted in cues previously paired with water/cocaine delivery.

**Results:**

The present study showed operant responses to produce novel sensory stimuli positively correlate with responding for cocaine during self-administration and during discrete cue-induced reinstatement, but no association with performance during extinction. A different pattern of associations was observed for a natural reward, in this case, water reinforcement. Here, the degree of novelty seeking also correlated with responding to water reinforcement and extinction responding; however, operant novelty seeking did not correlate with responding to water cues during testing of cue-induced reinstatement. Taken together, the incongruence of relationships indicates an underlying difference between natural and drug reinforcers.

**Conclusion:**

In summary, we found a reinforcer-dependent relationship between operant novelty seeking (i.e., sensation seeking) and responsivity to extinction and discrete cues signaling availability for cocaine (i.e., craving), demonstrating the validity of the operant novelty seeking model to investigate drug seeking and relapse.

**Supplementary Information:**

The online version contains supplementary material available at 10.1007/s00213-023-06441-4.

## Introduction

The US Department of Health and Human Services estimated that in 2019, approximately 5.2 million people used cocaine and 1.3 million met the criteria for a cocaine use disorder (SAMHSA n.d.). For those that achieve abstinence from cocaine, an estimated 25 (Simpson et al. 1999) to 45% (Carroll et al. 1993; Hall et al. 1991; McKay et al. 1999) relapse to using the drug within 1 year. Abstinence of longer than 1 year is reportedly achieved by as few as 5–15% of individuals (McCabe et al. 2018; White 2017). Together, these statistics indicate strong individual differences in vulnerability to drug addiction, and a subpopulation of individuals with a heightened risk of relapse (George and Koob 2017; Homberg et al. 2014; Le Moal 2009; Saunders et al. 2013; Sedighim et al. 2021). High individual variability coupled with the lack of available and effective treatment for cocaine use disorder highlights the need to identify factors, such as genetic and environmental variables, that can identify individuals with increased susceptibility to drug addiction and can potentially lead to therapies or strategies to attenuate the high rates of relapse and promote long-term recovery.

Interactions between genetic and environmental variables are often reflected in personality traits. One such trait of relevance to substance use disorders is the seeking of varied and novel experiences, referred to as sensation seeking (SS) (Zuckerman 1994). Sensation seeking correlates with aspects of drug addiction, including self-administration (SA) and acquisition (Cherpitel 1999; White 2017; Zuckerman 2007), such that individuals who score high on SS scales are more likely to abuse drugs than individuals with lower scores (Andrucci et al. 1989). There is strong evidence that sensation-seeking plays a role in various stages of the substance abuse continuum (Aklin et al. 2009; Castellani and Rugle 1995; Freund et al. 2021; Kahler et al. 2009; Kelly et al. 2006; McCabe et al. 2021; McKay et al. 1999; Nieva et al. 2011; Patkar et al. 2003, 2004; Regan et al. 2020; Tatari et al. 2021; Teichman et al. 1989), although see Ersche et al. (2010) and Murray et al. (2003). However, few studies have examined the relationship between sensation seeking and drug relapse (McKay et al. 1995, 1999), although Ramos-Grille et al. (2015) showed that sensation seeking predicts relapse to pathological gambling.

Sensation seeking has been modeled in animals by assessing locomotor reactivity to an inescapable novel environment or free-choice preference for novel objects/places (Bardo et al. 1996; Cain et al. 2005, 2006, 2008; Piazza et al. 1989; Robinet et al. 1998). Another model involves an operant procedure in which novel sensory stimuli are presented (Dickson and Mittleman 2020, 2021; Dickson et al. 2019; Gancarz et al. 2013). In this model of operant novelty seeking (ONS), the primary reinforcement is a response-contingent presentation of stimuli of moderate intensity that is not related to a biological need (Gancarz et al. 2013; Kish 1955, 1966; Lloyd et al. 2012a, b; Stewart 1960). We previously found that the procedures for ONS and locomotor responsivity to novelty have similar behavioral features: both procedures (i) produce within- and between-session habituation (Gancarz et al. 2011), (ii) result in increased responding after systemic administration of psychomotor stimulants (Gancarz et al. 2012b, c) and (iii) predict acquisition of methamphetamine SA (Gancarz et al. 2011). We also found that performance during operant sensory reinforcement positively associates with the number of cocaine infusions an animal self-administers (Gancarz et al. 2013).

While these earlier studies provided important insight regarding the relationship between sensory reinforcement and acquisition/early exposure to drug use, here, we sought to determine if operant novelty seeking predicts additional aspects of the addicted-like phenotype, such as relapse. Importantly, investigation of other animal models of response to novelty (e.g., locomotor, preference) has also focused on drug-taking behaviors with little research exploring how such measures would map onto addicted-like phenotypes (e.g., relapse). The goal of the present study was to determine whether ONS also predicts relapse of cocaine taking. We hypothesized that responding to the reinforcing effects of novel stimuli would predict cue-induced responding for cocaine but not for a natural reinforcer.

## Methods

### Subjects

Ninety male Sprague–Dawley laboratory rats (12 weeks old at the beginning of testing, 250–275 g, Charles River Laboratories) were tested. Rats were allowed to acclimate to the colony room for 2 days upon arrival. Behavioral testing occurred 5–7 days/week during the light phase of the 12-h light/12-h dark cycle. The rats were assigned to either cocaine (*n* = 42; experiment 1) or natural (water, *n* = 48; experiment 2) reinforcer groups. All rats had ad libitum access to food. Rats assigned to the water experiment were restricted to 30 min of water access a day for each phase of the experiment, which was provided in home cages following test sessions. Rats assigned to cocaine groups had ad libitum access to water. Rats were singly housed for the duration of the SA phase of the experiments to protect the catheter/harness assembly. This study was conducted in accordance with guidelines set forth by the Institutional Animal Care and Use Committee (IACUC; Protocol 15–04) at California State University, Bakersfield.

### Apparatus and drugs

#### Habituation/ONS

The experimental chambers had stainless-steel grid floors, aluminum front and back walls, Plexiglas sides, and a Plexiglas top. The chambers were 24 cm × 30 cm × 25 cm (inside dimensions). The right-side wall served as the intelligence panel, with two 3-cm-diameter circular snout poke holes (~ 2.5 cm above the floor grid) on either side of three LED lights (red, yellow, green; ~ 10 cm from the floor grid). A Sonalert was mounted on the back wall approximately 17 cm from the floor and 3 cm from the right side of the chamber. Snout/head entries into the snout poke holes were monitored and recorded with infrared detectors located 0.5 cm behind the front panel. All chambers were housed in sound-attenuating boxes. The entire apparatus was computer controlled through a MED Associates interface with MED-PC (version 5). The temporal resolution of the system was 0.01 s.

#### SA

Distinct operant chambers were used for SA and reinstatement with several important differences from those used for ONS. The two snout poke holes were water-dispensing receptacles (4 cm ´ 4 cm), and two additional stimulus lights were located above the receptacles. The stimulus lights were used as cues to indicate an infusion/reward was earned. The front right and left stimulus lights were equipped with a 28-V light bulb (SPC Technology, model #1819) with a white lens cap (Dialight, model #081–0135-303). A third stimulus light was in the middle of the back wall of the chamber, approximately 22 cm from the grid floor. Illumination of the back house light indicated the availability of the cocaine or water reinforcer. The house light was equipped with a 28-V light bulb (SPC Technology, model #1864) covered by a clear lens cap (Dialight, model #081–0135-303).

#### Drugs

(-)-Cocaine hydrochloride (gifted by the NIDA drug supply program) solutions were prepared weekly by dissolving the drug in sterile 0.9% saline at a concentration of 4.5 mg/mL. Pump duration was adjusted according to body weight every other day in order to deliver the correct dose of the drug (1.0 mg/kg/infusion, i.v.).

### Experiment 1: association between ONS and cocaine-seeking behavior

**Fig. 1:**
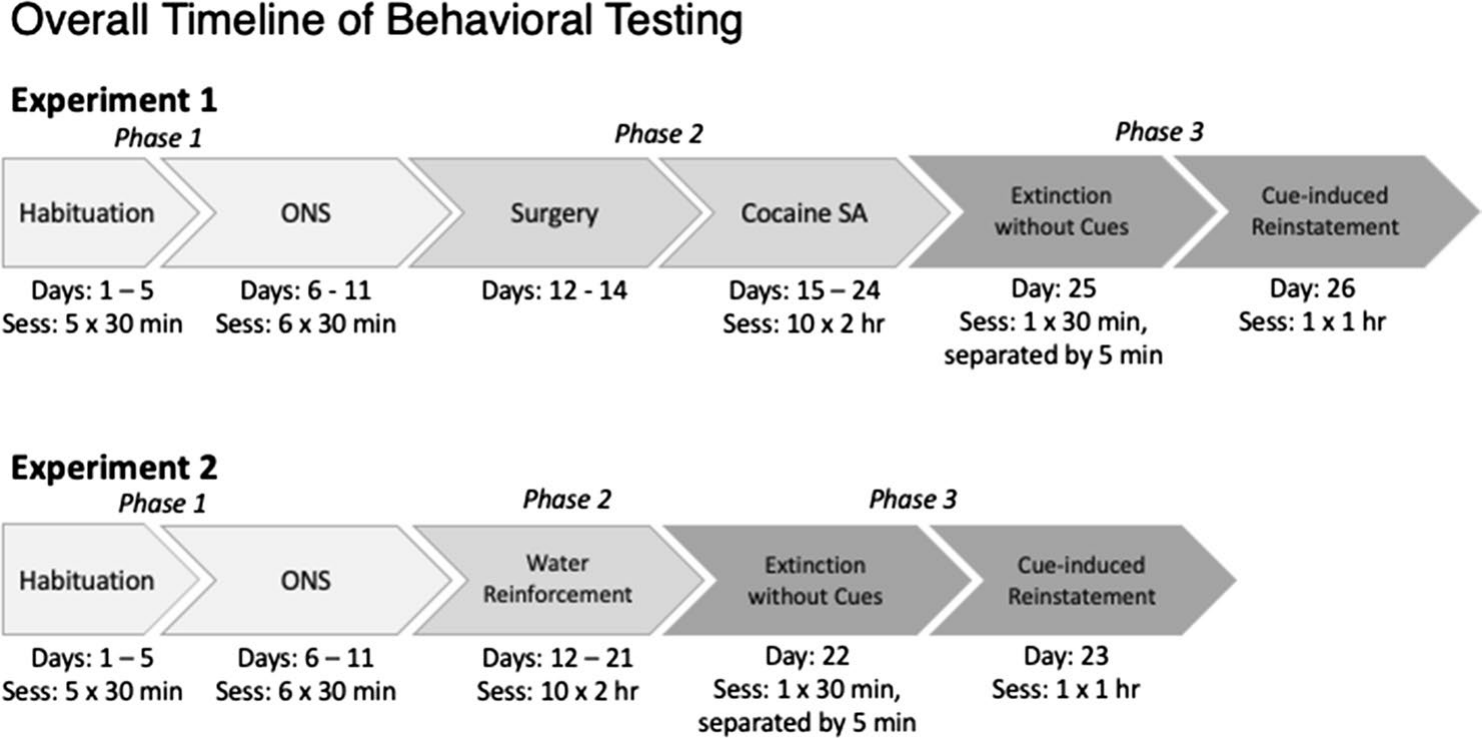
Illustration of experimental timeline for experiments 1 and 2

To examine the relationship between ONS and reinstatement to cocaine SA, a correlational research design was used with three phases (see Fig. 1, top). In phase 1, rats were habituated to dark experimental chambers and then tested for responding to novel operant sensory reinforcers. In phase 2, rats were tested for cocaine SA. In phase 3, rats were tested for extinction in the absence of discrete cues using a 1-day within-session extinction and then tested for cue-induced reinstatement the following day.

#### Habituation

Rats were introduced to the experimental chambers prior to testing to reduce potential confounds from exploratory or stress-related responding (i.e., to familiarize the rats with the chamber and decrease its novelty). Each day during the 5-day habituation phase, rats were placed in dark chambers for 30 min with no programmed stimuli: snout poke responses were recorded but did not produce any programmed consequences.

#### ONS

Following habituation, animals were placed in the experimental chambers for six 30-min test sessions to measure ONS as previously described with a few modifications (Gancarz et al. 2012c). During this phase, the chambers were dark except when response-contingent visual and auditory stimuli were presented. Reinforced active responses produced a complex visual/auditory stimulus, which consisted of the illumination of three LED lights (yellow, red, and green; illumination duration varied between each presentation from 2 to 8 s) and the presentation of an auditory stimulus (activation of Sonalert of between 1 and 3 s) on a variable interval 1-min schedule of reinforcement (the average of every minute an active response was reinforced with any combination of the available stimuli). The stimuli were presented in random sequences and combinations to prolong their novelty. Responses to the inactive alternative had no programmed consequences.

#### Cocaine SA

Following ONS, rats were implanted with jugular vein catheters, maintained, and tested for patency as previously described (Gancarz et al. 2012b), and only rats with patent catheters were used in the study. After recovery, the rats were connected to the operant chamber as previously described and tested for cocaine SA (2-h sessions for 10 days). During SA sessions, responses to the active snout poke were reinforced with an infusion of 1.0 mg/kg cocaine according to a Fixed Ratio 3 (FR3) schedule of reinforcement. Reinforced responses resulted in the delivery of drug infusion and the illumination of the cue light above the snout poke receptacle, indicating drug dispersal. Following drug delivery, the houselight was extinguished and the chamber was dark for a 30-s time-out period. Re-illumination of house light following the timeout period then indicated the availability of the cocaine reinforcer.

#### Extinction responding in the absence of the discrete cue

Twenty-four hours following the last SA session, subjects were tested for extinction responding in the absence of the discrete cue and underwent a one-day multiple within-session extinction as previously described (Gancarz et al. 2015; Gancarz-Kausch et al. 2014). Briefly, rats were returned to the operant chambers where cocaine testing occurred. In this phase, chambers were dark, and responses were recorded but resulted in no programmed consequences. Stimuli previously paired with cocaine infusions were no longer available, and the catheter/harness assembly was connected to a saline solution (no infusions occurred).

The rats were allowed to respond for a minimum of twelve 30-min sessions to ensure the behavior was thoroughly extinguished (sessions separated by 5-min intervals, during which rats were removed from the operant chamber and returned to the home cage until the next session began) and a maximum of 16 sessions, or until their responses fell to < 20 snout pokes per session. The first session was used as a test of extinction responding in the absence of the discrete cue (Crombag et al. 2008).

#### Discrete cue-induced reinstatement

The test for discrete cue-induced reinstatement occurred the following day, during which subjects were returned to the operant chambers for 1 h. The catheter/harness assemblies were connected to a saline solution, but no drug was delivered (Gancarz et al. 2015). Snout poke responses produced cues previously paired with drug delivery.

### Experiment 2: association between ONS and water-seeking behavior

The same correlational research design was used to examine the relationship between ONS and reinstatement to a natural reinforcer, in this case, water (Fig. 1, bottom). In phase 1, rats were first habituated to dark experimental chambers and then tested for responding to novel operant sensory stimuli. In phase 2, rats were tested for water SA. In phase 3, rats underwent *extinction responding in the absence of the discrete cue* using a 1-day within-session extinction and were tested for cue-induced reinstatement the following day.

#### Habituation and ONS

Parameters were identical to those described in experiment 1.

#### Water SA

Water reinforcement was conducted for 10 days in 2-h sessions. Responses to the active snout poke were reinforced with a delivery of 30 μL of water directly into the water receptacle. All other parameters were identical to those conducted for cocaine SA. After the session each day, the rats were returned to the colony and given 30 min access to water.

#### Extinction and cue-induced reinstatement

Parameters were identical to those described in experiment 1.

### Data analysis

The primary measures of ONS were the number of responses to the active and inactive snout pokes (active responses and inactive responses, respectively) and the number of sensory rewards earned. The dependent variables used to measure responding for water and cocaine reinforcers were the same as those used for ONS (numbers of active and inactive responses and numbers of cocaine infusions and water presentations). The dependent variable used to measure responding for extinction responding in the absence of discrete cues and cue-induced reinstatement was the number of active responses. Percent change in responding was used to normalize performance to determine relative changes in performance between days of testing, calculated as follows: [(day A – day B)/day B] × 100. Using this calculation, positive values indicate an increase in responding, whereas negative values indicate a decrease in responding from day B to day A. Analyses of variance (ANOVAs) were used where appropriate to compare experimental groups.

Pearson’s correlation tests were used to determine the relationships between (i) ONS and cocaine or water SA, (ii) ONS and extinction responding in the absence of discrete cues, and (iii) ONS and cue-induced reinstatement (active responses for cocaine/water). A two-tailed alpha of < 0.05 indicated a significant association. All dependent variables for the cocaine SA and water reinforcement were collapsed across all 10 days of testing for the correlation analysis. Dependent variables for ONS were averaged across the last 3 days (days 4–6) of ONS testing.

An "extreme groups" approach was also used, in which animals that scored in the highest and lowest quartiles with respect to ONS were compared. Animals with ONS scores in the top quartile were classified as high-ONS (experiment 1: *n* = 10; experiment 2: *n* = 12) and animals with scores in the lowest quartile were classified as low-ONS (experiment 1: *n* = 10; experiment 2: *n* = 12). Data from animals in the middle half were not used for this analysis. The extreme groups approach has been used in research investigating individual differences in sensation/novelty-seeking to predict the acquisition of SA (Piazza et al. 1989, 1990, 2000). An independent samples *t* test was used to compare the dependent measures defined above. An alpha of < 0.05 used to identify a significant difference.

## Results

### Experiment 1: ONS performance

#### Habituation and ONS

**Fig. 2:**
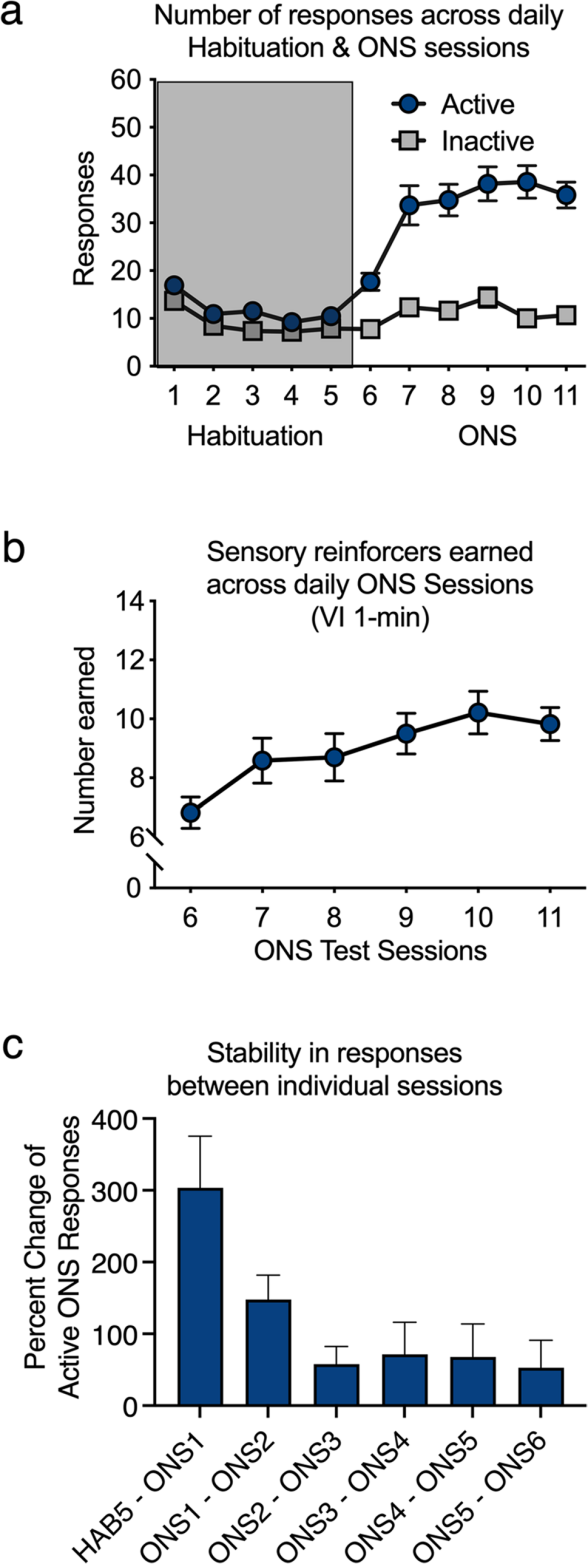
Performance on ONS. (**a**) Performance during the habituation phase is shown in the shaded region, and performance in the ONS phase is shown on the right. (**b**) Numbers of sensory reinforcer presentations earned across sessions 6–11 of ONS. (**c**) Percent change in performance between days of testing. Data are averages (± SEM); *n* = 42

A one-way within-subject ANOVA revealed a significant main effect of the session on the total number of snout pokes (responses) during the habituation phase [F(4,164) = 11.324, *P* < 0.001]. These data indicate that rats decreased responding as the context of the experimental chamber became familiar (Fig. 2a).

A one-way within-subject ANOVA revealed a significant main effect of session on the number of active responses during ONS testing [*F*(5, 205) = 8.977,* P* < 0.01] (Fig. 2a). A one-way ANOVA with sessions as the within-subject factor revealed a significant main effect of time on the number of sensory reinforcers earned [*F*(5, 205) = 4.782,* P* < 0.02] (Fig. 2b). Importantly, when comparing the numbers of active and inactive responses during ONS testing, follow-up paired-samples *t* tests revealed significantly more active responses on days 1 through 6 [1: *t*(45) = 5.454*, P* < 0.05; 2: *t*(45) = 5.466*, P* < 0.05; 3: *t*(45) = 6.859,* P* < 0.05; 4: *t*(45) = 5.993,* P* < 0.05; 5: *t*(45) = 8.230,* P* < 0.05; and 6: *t*(45) = 8.627,* P* < 0.05]. These data indicate the rats responded more to the active snout poke, which produced the response-contingent sensory reinforcers.

Sensation seeking has previously been shown to be stable across time (Lynne-Landsman et al. 2011; Pedersen 1991; Zuckerman 2007; Zuckerman et al. 1980); thus, we assessed the percent change in active responses throughout the ONS testing phase. There was a dramatic change in responding between day 5 of habituation and day 1 of ONS, demonstrating that the contingent sensory stimuli were reinforcing (Fig. 2c). The percent change in responding became progressively smaller between days 1 and 2 of ONS and between days 2 and 3 but was maintained thereafter, reflecting the stability of the behavior.

#### Cocaine SA and cue-induced reinstatement

**Fig. 3:**
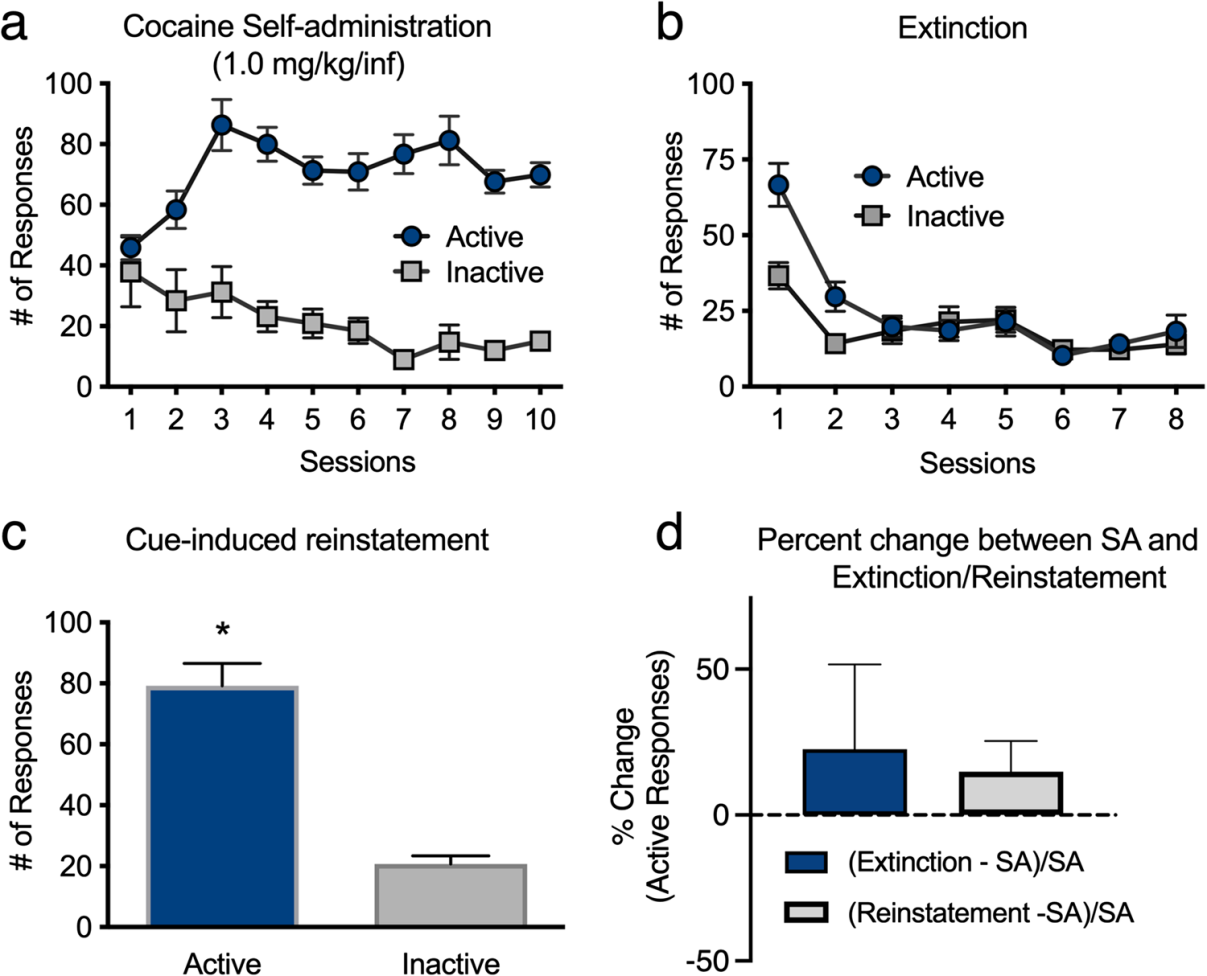
Performance during cocaine SA. **a** Total numbers of inactive responses and active responses for 1.0 mg/kg/infusion cocaine. **b** Total number of inactive responses and responses to the previously active snout poke during extinction. **c** Numbers of active and inactive responses during the 1-h test of cue-induced reinstatement. **d** Percent change of active responses from cocaine SA to Extinction (Ext) and Cue-induced Reinstatement (Reins). Data are averages (± SEM). *P < 0.05

A one-way ANOVA with SA sessions as the within-subject factor showed a significant main effect of session on the number of active responses during cocaine SA testing [*F*(9,369) = 6.450,* P* < 0.02] (Fig. 3a). A follow-up paired-sample *t* test revealed that rats responded significantly more on day 10 than on day 1 of cocaine SA [*t*(41) = -5.078,* P* < 0.01], indicating the acquisition of responding for cocaine. No significant main effect of inactive responding was detected. The numbers of cocaine infusions earned were also stable across days of testing (day 1: $\overline{\text{X}}$ = 17.50 ± 0.69 [cumulative dose of 17.50 ± 0.69 mg/kg]; day 10: $\overline{\text{X}}$= 18.45 ± 0.57 [cumulative dose of 18.45 ± 0.57 mg/kg]) (Supplemental Fig. 1a).

A one-way ANOVA with extinction sessions as the within-subject factor showed a significant main effect of the session on the total number of responses [*F*(7,287) = 23.932,* P* < 0.001]. A follow-up paired-sample *t* test revealed the rats emitted significantly more active responses during extinction session 1 than during extinction session 8 [*t*(41) = 7.948,* P* < 0.01], indicating extinction had occurred (Fig. 3b). A within-subject *t* test revealed rats emitted significantly more active responses than inactive responses during the testing of cue-induced reinstatement [*t*(41) = 8.594,* P* < 0.01] (Fig. 3c). These data indicate that the cues previously paired with cocaine elicited drug-seeking behavior.

There was a positive percent change from active responses emitted on Day 10 of cocaine SA to active responding during extinction and cue-induced reinstatement (Fig. 3d). These data indicate that rats made more active responses during tests of Extinction Session 1 and cue-induced reinstatement compared to day 10 of cocaine SA.

#### ONS is associated with cocaine SA, extinction, and cue-induced reinstatement

**Fig. 4:**
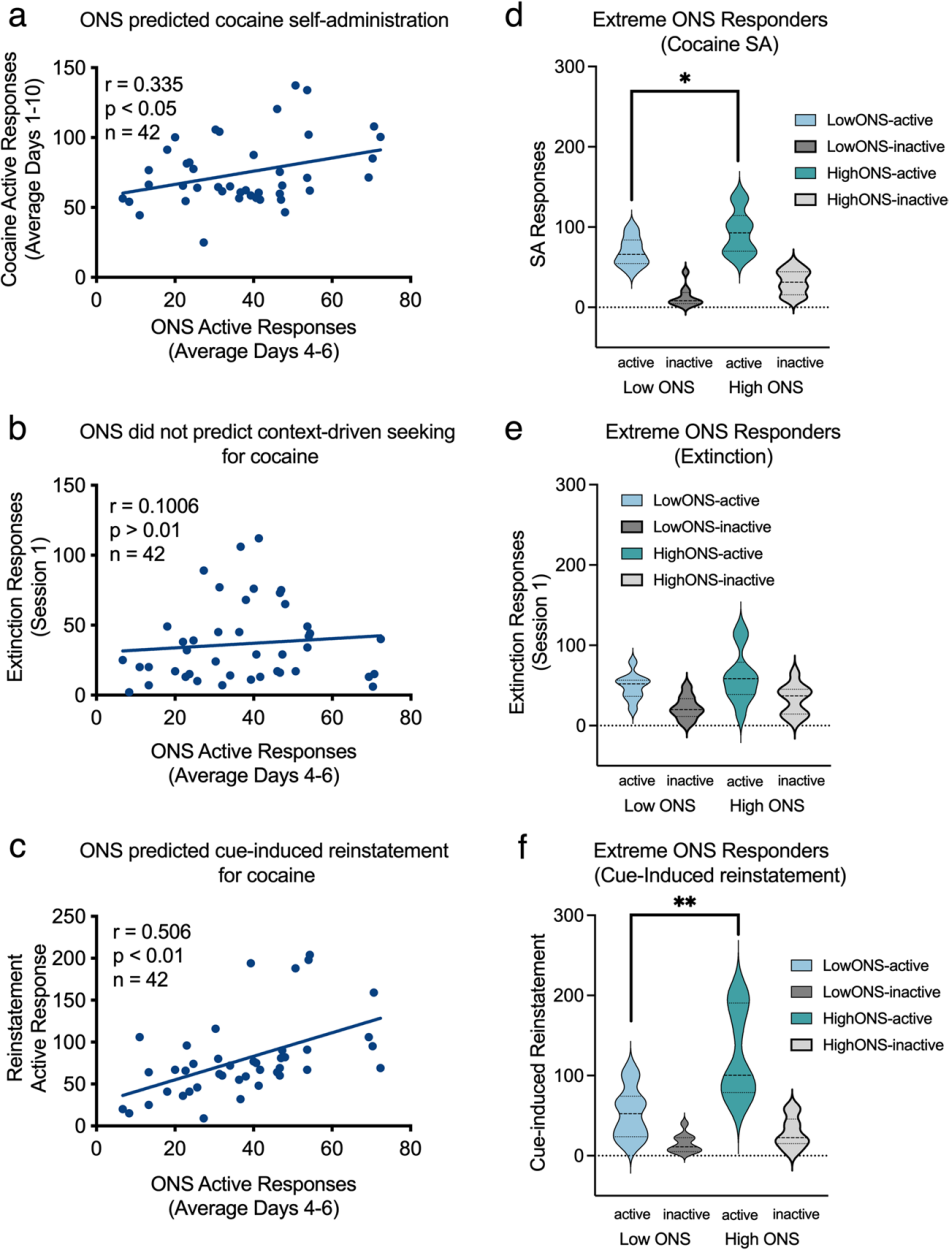
Correlations between ONS and cocaine SA. Relationships between measures of ONS (average across days 4–6) and: **a** active responses to receive an infusion of 1.0 mg/kg cocaine (averaged across days 1–10), **b** responses emitted during extinction (session 1), and **c** responses to cues previously signaling delivery of cocaine. Performance of top quartile (high-ONS, *n* = 10) and the bottom quartile (low-ONS, *n* = 10) rats for novelty seeking on **d** number of responses emitted during cocaine SA (average days 1–10), **e** extinction responding (session 1), and **f** cue-induced reinstatement. *P < 0.05; **P < 0.01

We sought to determine the association between ONS and cocaine self-administration. Therefore, we averaged performance on operant novelty seeking on days 4–6 of testing when behavior was stable (Fig. 2c). The average numbers of active responses each rat emitted on days 4–6 during ONS positively correlated with the average numbers of active responses (averages from days 1 to 10) emitted during cocaine SA (*r* = 0.34,* P* < 0.05) (Fig. 4a). However, active ONS responses did not predict the number of cocaine infusions (see Table 1) because they were limited to 20 infusions per session (active responses were still recorded during drug time-out periods). The number of inactive responses during ONS did not correlate with the number of sensory reinforcers, cocaine infusions earned, or active responses (Table 1). ONS also did not correlate with performance during extinction session 1 (Fig. 4b). Interestingly, however, the number of active responses during ONS also positively correlated with the numbers of active responses during cue-induced reinstatement (*r* = 0.51,* P* < 0.05) (Fig. 4c). There were no correlations between inactive responses during ONS and the numbers of ONS sensory reinforcers earned or with any of the other dependent measures of cue-induced reinstatement.

**Table 1:** Correlational matrix for experiment 1 (*n* = 42)

Phase	Variable	ONS^a^			Cocaine SA^b^			Cue-induced active responses
		Active responses	Inactive responses	Reinforcers earned	Active responses	Inactive responses	Reinforcers earned	
ONS^a^	Active responses	-	0.126	0.745**	0.335*	0.121	0.143	0.506**
	Inactive responses		-	0.202	− 0148	0.309*	0.115	− 0.054
	Reinforcers earned			-	0.225	− 0.065	0.179	0.318*
Cocaine SA^b^	Active responses				-	0.091	0.477**	0.366*
	Inactive responses					-	− 0.025	− 0.073
	Reinforcers earned						-	0.246
Cue-induced active responses								-

^a^Performance was averaged across days 4–6 of testing

^b^Performance was averaged across days 1–10 of testing

^*^P < 0.05 **P < 0.01

Animals were sorted based on the number of active responses emitted during ONS and the highest (high-ONS) and lowest (low-ONS) quartiles were compared on cocaine self-administration (average days 1–10) using an independent samples t-test. High-ONS rats emitted significantly more active [*t*(18) = 2.384, *P* < 0.05] but not inactive responses during cocaine SA than rats classified as low-ONS (Fig. 4d). There was no significant difference between high-ONS or low-ONS in the number of active and inactive responses emitted during a test of extinction (session 1; Fig. 4e). High-ONS rats also had significantly more active [*t*(18) = 3.508, *P* < 0.05] but not inactive responses than their low-ONS counterparts during the test of cue-induced reinstatement (Fig. 4f). Taken together, the data suggest that ONS predicts responding for cocaine during SA and cue-induced reinstatement, but not extinction responding in absence of discrete cues.

### Experiment 2: ONS performance

#### Habituation and ONS

**Fig. 5:**
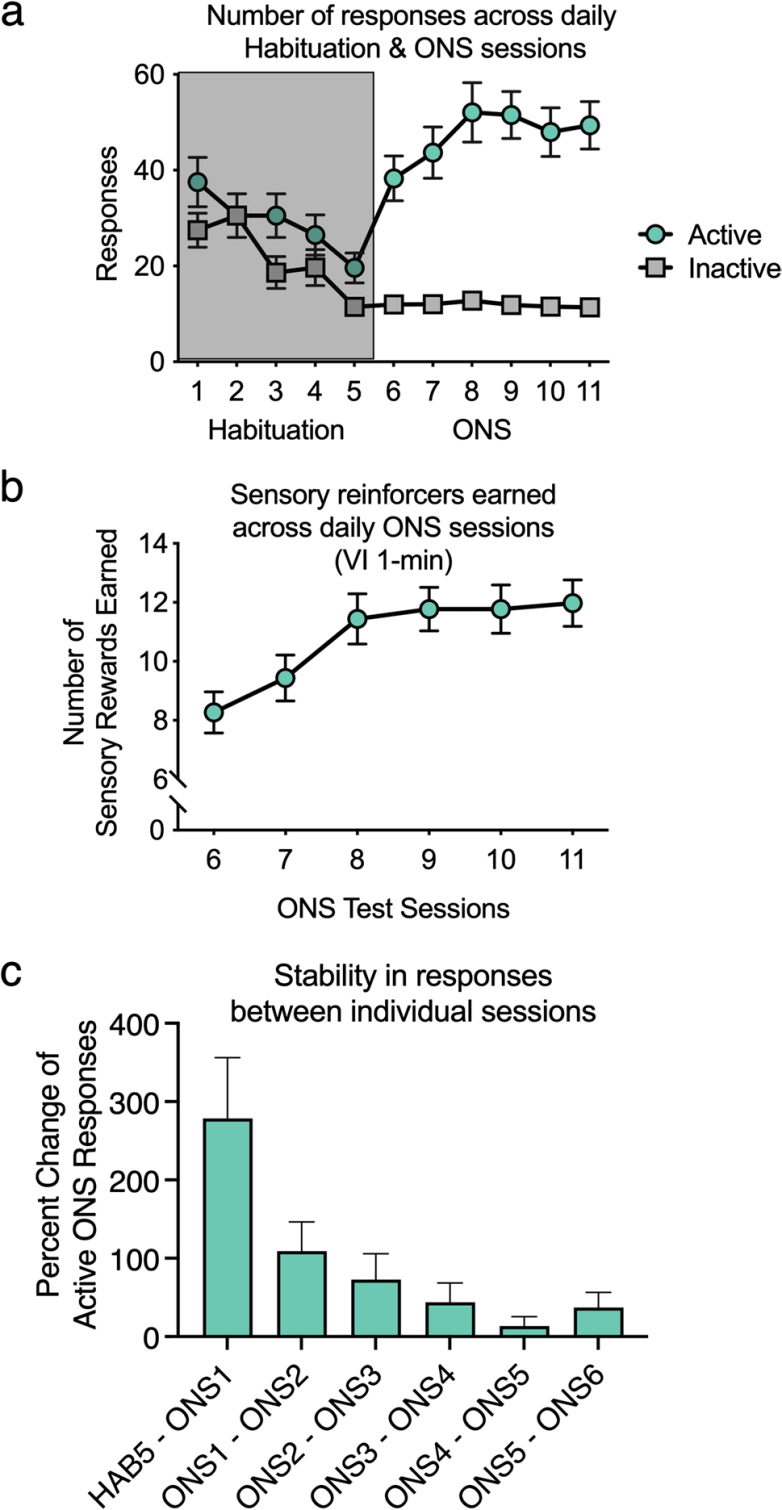
Performance on ONS. **a** Performance during the habituation phase is shown in the shaded region, and performance in the ONS phase is shown on the right. **b** Numbers of sensory reinforcer presentations earned across sessions 6–11 of ONS. **c** Percent change in performance between days of testing. Data are averages (± SEM); *n* = 48

A one-way within-subject ANOVA revealed a significant main effect of the session on the total number of responses during the habituation phase [*F*(4, 188) = 9.197, *P* < 0.001], indicating that the rats in this cohort also became familiar with the chamber context (Fig. 5a).

A one-way within-subject ANOVA revealed a significant main effect of session on the number of active responses made during ONS testing [*F*(5, 235) = 2.530,* P* < 0.05] (Fig. 5a). Furthermore, a one-way ANOVA with sessions as the within-subject factor revealed a significant main effect of time on the number of sensory reinforcers earned [*F*(5, 235) = 8.154,* P* < 0.001] **(**Fig. 5b). These data from a separate cohort of rats demonstrate the reliability of the reinforcing effects of the response-contingent sensory stimuli used in our experiments.

Similar to that observed with the cohort of rats in experiments 1, the percent change in active responding was highest between the last day of habituation and the first day of ONS and then decreased thereafter, indicating a stabilization in performance (Fig. 5c).

#### Water reinforcement and cue-induced reinstatement

**Fig. 6:**
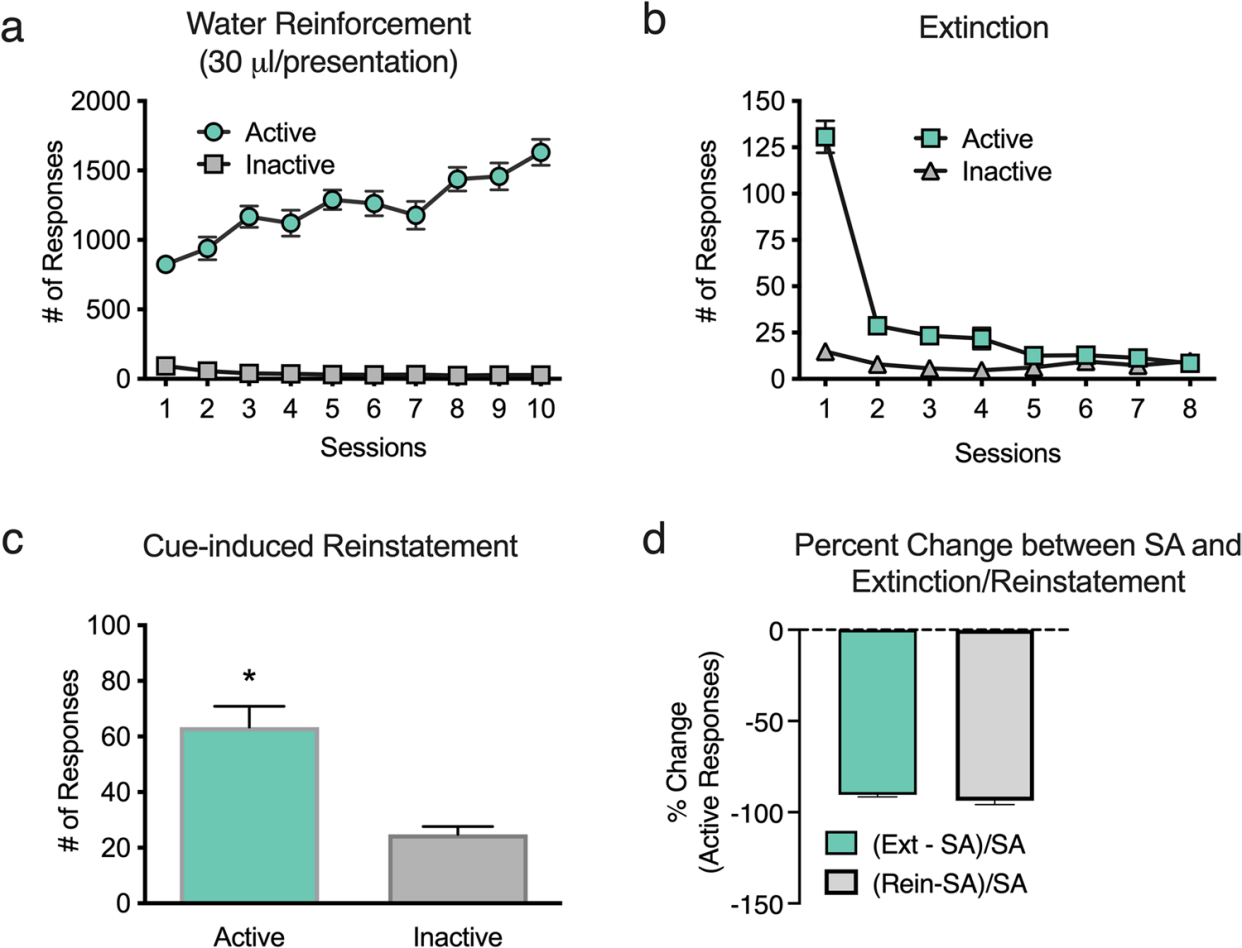
Performance during water reinforcement. **a** Total numbers of inactive responses and active responses for delivery of 30 μL water. **b** Total number of inactive responses and responses to the previously active snout poke during extinction. **c** Numbers of active and inactive responses during the 1-h test of cue-induced reinstatement. **d** Percent change of active responses from water SA to extinction (Ext) and cue-induced reinstatement (Reins). Data are averages (± SEM). *P < 0.05

A one-way ANOVA with sessions as the within-subject factor showed a significant main effect of session on active responses for a water reinforcer [*F*(9,423) = 14.208,* P* < 0.01] (Fig. 6a). A follow-up paired-samples *t* test revealed that rats made significantly more responses on day 10 than on day 1 of water reinforcement [*t*(47) =  − 10.352,* P* < 0.01], indicating the acquisition of responding for water. Consistent with this, a one-way ANOVA with sessions as the within-subject factor showed a significant main effect of the session on the number of water rewards earned [*F*(9,423) = 8.717,* P* < 0.01] (Supplemental Fig. 1b). A follow-up paired-sample *t* test revealed that rats earned significantly more water on day 10 than on day 1 of water reinforcement [*t*(47) =  − 10.352,* P* < 0.01]. A one-way ANOVA with water reinforcement sessions as the within-subject factor also showed a significant main effect of session on inactive responses [*F*(9,423) = 26.849,* P* < 0.01) (Fig. 6a), with follow-up paired-sample *t* tests indicating rats made significantly more inactive responses on day 1 than on day 10 [*t*(47) = 7.959,* P* < 0.01]. Taken together, these data indicate acquisition of water reinforcement occurred across days of testing in thirsty rats.

A one-way ANOVA with extinction sessions as the within-subject factor showed a significant main effect of the session on total active responses for water reinforcement [*F*(7,329) = 103.625,* P* < 0.001]. A follow-up paired-sample *t* test revealed significantly more active responses during session 1 than during session 8 [*t*(47) = 13.825,* P* < 0.01], indicating the reinforced behavior had been extinguished (Fig. 6b). A within-subjects *t* test revealed that rats made significantly more active responses than inactive responses during the test of cue-induced reinstatement [*t*(47) = 4.64,* P* < 0.01) (Fig. 6c), indicating that the cues previously paired with water elicited seeking behavior.

Contrary to the pattern of results for cocaine in experiment 1, there was a negative percent change for both extinction (session 1) and cue-induced reinstatement from performance on the last day of water reinforcement. These data indicate that rats made fewer active responses during extinction (session 1) and cue-induced reinstatement compared to day 10 of water reinforcement (Fig. 6d).

#### ONS is associated with water reinforcement but not cue-induced reinstatement of water-seeking

**Fig. 7:**
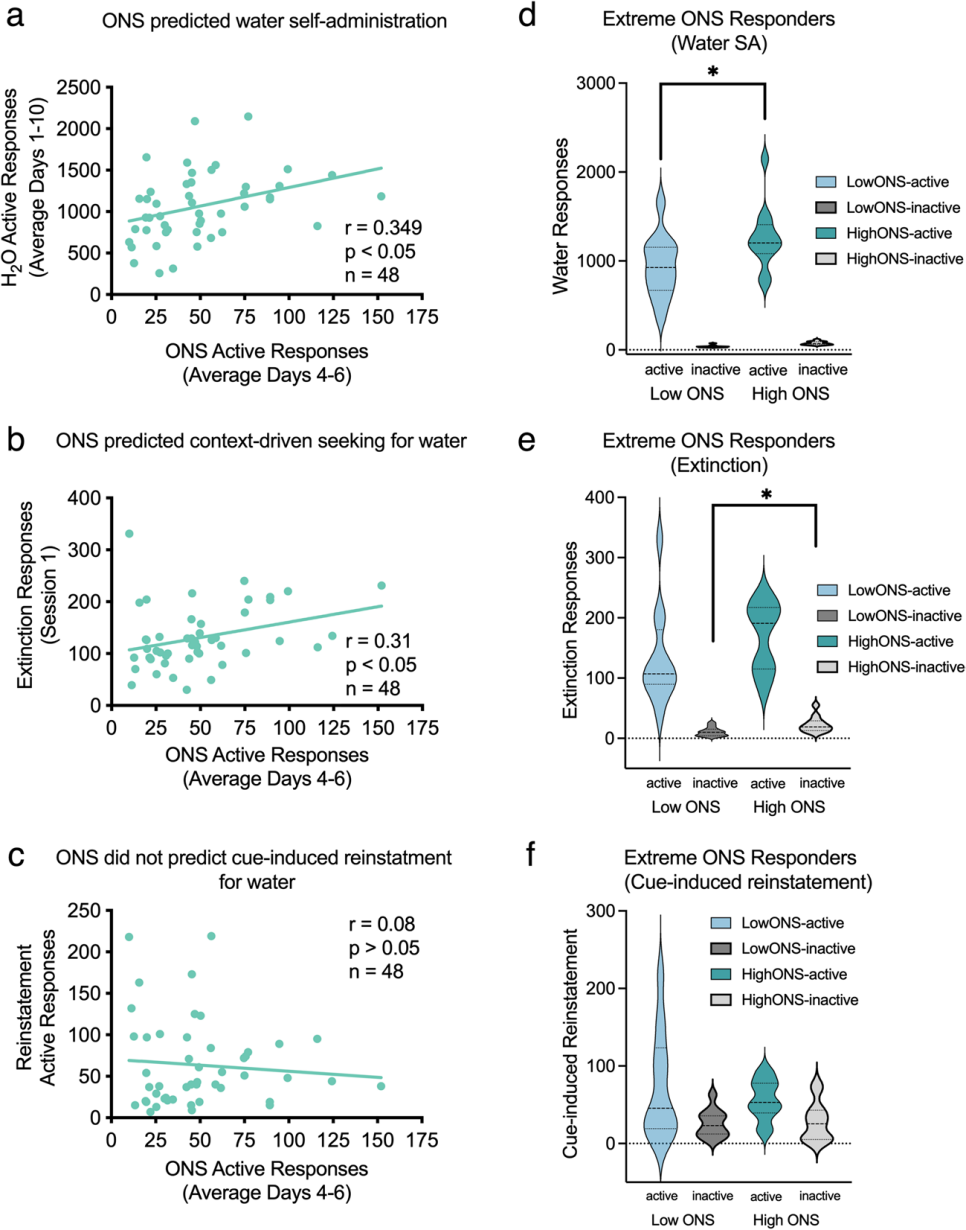
Correlations between ONS and water SA. Relationships between measures of ONS (average across days 4–6) and **a** active responses to receiving a delivery of 30 μL water (averaged across days 1–10), **b** and **c** responses to cues previously signaling delivery of water. Performance of top quartile (high-ONS, *n* = 10) and the bottom quartile (low-ONS, *n* = 10) rats for novelty seeking on **d** number of responses emitted during water SA (average days 1–10), **e** extinction responding (session 1) and, and **f** cue-induced reinstatement of water seeking. *P < 0.05; **P < 0.01

Table 2 shows a correlational matrix for the relationships between ONS testing (average from days 4–6 for each animal) and water reinforcement testing (averages from days 1–10 for each animal). The number of ONS active responses positively correlated with the number of active responses for water reinforcement (*r* = 0.35,* P* < 0.01) (Fig. 7a). The number of ONS active responses also predicted the number of water reinforcers earned (*r* = 0.30,* P* < 0.01). Interestingly, there was a significant positive association between ONS active responses and responses emitted during extinction (session 1; Fig. 7b). However, there was no correlation between active responses for ONS and those for cue-induced reinstatement (Fig. 7c).

**Table 2:** Correlational matrix for experiment 2 (*n* = 48)

Phase	Variable	ONS^a^			Water SA^b^			Cue-induced active responses
		Active responses	Inactive responses	Reinforcers earned	Active responses	Inactive responses	Reinforcers earned	
ONS^a^	Active responses	-	0.447**	0.905**	0.349*	0.500**	0.304**	− 0.089
	Inactive responses		-	0.338*	0.255	0.494*	0.262	0.137
	Reinforcers earned			-	0.342*	0.348*	0.341*	− 0.136
Water SA^b^	Active responses				-	0.511*	0.787**	0.172*
	Inactive responses					-	0.295*	0.038
	Reinforcers earned						-	0.279
Cue-induced active responses								-

^a^Performance was averaged across days 4–6 of testing

^b^Performance was averaged across days 1–10 of testing

^*^P < 0.05 **P < 0.01

Using the extreme groups approach, independent-sample *t* tests showed that rats classified as high-ONS made significantly more active [*t*(22) =  − 2.195, *P* < 0.05] but not inactive responses than their low-ONS counterparts during water reinforcement testing (Fig. 7d). High ONS rats emitted significantly more inactive responses [*t*(22) =  − 2.756, *P* < 0.05 and a trend for more active responses (*P* = 0.1) than low ONS rats during a test of extinction responding in absence of discrete cues (Fig. 7e). However, there were no significant differences between the high- and low-ONS rats during a test of cue-induced reinstatement (Fig. 7f). Taken together, these data suggest ONS predicts responding for water reinforcers and extinction responding in absence of discrete cues but not cue-induced reinstatement of the behavior.

## Discussion

The primary aim of this study was to examine the association between an animal model of sensation seeking and responsiveness for extinction responding in the presence and absence of discrete cues paired with cocaine and water reinforcers in adult male rats. We found a significant positive association between ONS performance and active responding in both cocaine self-administration and water reinforcement. Our data also demonstrate an animal’s level of responding to novel sensory stimuli predicts the propensity to seek discrete cues paired with cocaine use, but not extinction responding in the absence of discrete cues. These findings suggest that novelty or sensation seeking, as a measurable personality trait, may serve to predict individual responses that are associated with substance use disorder and relapse.

Our results replicate and extend those from studies showing that sensory reinforcement predicts responding to drugs and natural rewards (Gancarz et al. 2011, 2013; Mitchell et al. 2005). For example, we previously showed that responding to a visual stimulus reinforcer correlates with the number of cocaine infusions animals will self-administer (Gancarz et al. 2013). The present study utilized different experimental parameters, such as a higher dose of cocaine (1.0 mg/kg/inf), which has been shown to be more likely to support acquisition (Carroll and Lac 1997; Gancarz et al. 2013; Kosten et al. 1997), and increased response requirement per reward, illustrating a robust relationship between novelty seeking and cocaine SA. Taken together, these experiments show that ONS is a reliable animal model of sensation seeking and is associated with drug-taking behavior.

The present study is among the first to demonstrate the association between sensation seeking and the propensity to relapse to drug use. Whereas the readiness to self-administer cocaine is thought to reflect the sensitivity of an animal to the rewarding properties of the drug, the reinstatement of responding when a drug-paired cue is presented is thought to reflect the vulnerability to relapse. Our data suggest that an animal that finds novel stimuli highly rewarding is more likely to resume behaviors that were previously linked with a drug reward. However, the reinstatement of seeking behaviors did not extend to a natural reinforcer, in this case, water delivery to water-restricted rats. Although visual stimuli were used both as a reinforcer (in the ONS task) and as a cue associated with reinforcers (cocaine and water), the level of responding for the visual stimuli did not correlate with the reinstatement of responding for both reinforcers (i.e., only with the reinstatement of drug seeking). Thus, the rats did not generalize the reinforcement and cue properties of the visual stimuli.

A different pattern emerged when testing extinction responding (when rats were re-exposed to the chambers in the absence of cue or reinforcer availability). Here, a positive association was observed between ONS and rats previously trained to respond to water reinforcers, but no relationship was observed between ONS and performance in rats previously trained to respond to cocaine. Taken together, these data illustrate a different pattern of relationships between ONS and extinction and discrete cue-seeking for water and cocaine reinforcers. Rats responding to cocaine showed a relationship to discrete cues but no relationship to extinction, whereas rats responding to water showed a relationship to extinction rather than discrete cues. From an ethological perspective, water itself is a discrete cue and encompasses a distinct motor and oral consummatory response (e.g., licking, swallowing). In contrast, cocaine as a stimulus is more perceptually diffuse and relies on interoceptive perceived response. This inherent difference in the basic properties of the reinforcer/nature of the stimuli and consummatory behavior may result in differences in associations made in guiding behavior. Overall, ONS similarly predicts the sensitivity to reinforcers but differentially predicts the seeking of those reinforcers.

The seeking of natural reinforcers (e.g., food or water) is driven by an internal state, whereas the seeking of drugs is driven by many factors. We can begin to differentiate these by the responses for a given reinforcer. In the present study, the level of responding was markedly higher for water than cocaine, in part because of the experimenter-imposed parameters (e.g., limit in the number of available cocaine infusions to prevent overdose, economy type). These constraints aside, variances in operant response to drug and non-drug rewards have been fully characterized (Gancarz et al. 2012b; Kearns 2019; N Kearns et al. 2011). For example, Christensen et al. (2009) showed less elasticity for food than cocaine in a demand analysis for both Fisher and Lewis rats. Differences in reinstatement performance for drug and natural rewards have also been reported. Ahmed and Koob (1997) showed rats exhibited stress-induced reinstatement for cocaine but not food. Furthermore, while incubation of cue-induced reinstatement has been demonstrated for both sucrose and cocaine, greater responding was reported for the drug (Li and Frantz 2010; Lu et al. 2004). Given the distinct behavioral patterns between these two reinforcer types, it would be of interest to determine if sensory reinforcement would predict the temporal profile of relapse-like behavior, such as the progressive enhancement of relapse behaviors across time (i.e., incubation, Grimm et al. 2001).

Another difference in responding between reinforcer types was revealed when considering the percent change between the last day of reinforcement testing and relapse. Whereas responding for cocaine was higher during reinstatement than on the last day of SA, responding for water was much lower than it was during SA. However, the patterns of extinction of responding for both reinforcers were similar. Our data suggest that although the same cues can drive behaviors to obtain natural and drug rewards, the strength of these cues differs depending on the type of reward. Indeed, the importance of cues in cocaine SA has been well established (Schenk and Partridge 2001). The stronger cue-induced reinstatement response for cocaine may reflect incentive salience, in which cues gain motivational incentive value (Flagel et al. 2009).

There is evidence that sensation seeking is more strongly associated with craving for cocaine and other stimulants (Gancarz et al. 2011, 2012a, c, 2013; Lloyd et al. 2012a, b; Walsh et al. 2010) than for cigarette smoking (Billieux et al. 2007; Doran et al. 2009; Reuter and Netter 2001) and alcohol consumption (Flaudias et al. 2019). Sensation seeking has also been implicated in relapse to cocaine use (McKay et al. 1995, 1999), cigarette smoking (Kahler et al. 2009), and alcohol (Kravitz et al. 1999; Marra et al. 1998; Meszaros et al. 1999; Müller et al. 2008) in humans. Recently, Xu et al. (2022) showed that high levels of sensation seeking reduced the motivation to remain abstinent from drug use. However, more definitive studies investigating the interaction between sensation seeking and craving/relapse are necessary.

As demonstrated here, ONS presents a valid model to explore the associations between sensation seeking and craving for reinforcers in males. The focus of this study was to examine the role of novelty seeking as a predictor and or correlate to drug-seeking behavior following exposure to cocaine. As novelty-seeking has been shown to be a sexually dimorphic trait (Cross et al. 2013; Obst et al. 2021; Zuckerman et al. 1978), we chose to analyze this relationship in male rats. Ongoing studies will determine whether this procedure predicts these same relationships in females. Furthermore, research has extensively explored animal models of sensation seeking and response to cocaine, *d*-amphetamine, and methamphetamine (Gancarz et al. 2011, 2012a, c, 2013; Lloyd et al. 2012a, b); however, it is unclear if these relationships are unique to psychomotor stimulants or can be generalized to other drugs of abuse. Indeed, there is some evidence of divergent relationships between sensation seeking and other drug classes. For example, Galizio and Stein (1984) showed sensation seeking was more strongly associated with polydrug use than opiate and depressants use alone in humans. Future research is needed to explore the relationship between operant novelty seeking and other drugs of abuse.

Other studies have shown a relationship between sensation seeking and enhanced responses to other non-drug rewarding stimuli in humans. For example, high sensation seekers have altered food preferences (Logue and Smith 1986; Terasaki and Imada 1988), elevated levels of gambling (Coventry and Brown 1993), and operant responses to monetary reinforcers (Bornovalova et al. 2009). To the best of our knowledge, no studies have investigated the role of sensation seeking in craving for natural rewards, such as food. The lack of research in this area highlights the key importance in the predictive validity of animal models. The present study is significant because it shows the predictive ability of ONS as a model of sensation seeking and that it could be used to examine the vulnerability to a variety of drug and non-drug reinforcers.

## Conclusion

Our results indicate a reinforcer-dependent relationship between ONS and responsivity to cues signaling the availability of cocaine and water reinforcers. ONS correlated with cue-induced reinstatement to cocaine, an animal model of relapse; however, ONS was not associated with cue-induced reinstatement for water reinforcers. These data support our hypothesis that sensitivity to the reinforcing effects of novel visual and auditory stimuli predicts relapse as well as other drug-taking behaviors. Thus, ONS is an animal model of sensation seeking that can be used to predict drug craving and relapse to cocaine. Future studies are needed to determine if sensation seeking similarly predicts the use of other drugs in males and females.

## Supplementary Information

Below is the link to the electronic supplementary material.

Supplementary file1 (DOCX 83 KB)

## Data Availability

The datasets generated and/or analyzed during the present study are available from the corresponding author upon reasonable request.

## Abbreviations

ONS: Operant novelty seeking
SA: Self-administration
Ext: Extinction
Reins: Reinstatement
SS: Sensation seeking

## References

[CR1] Ahmed SH, Koob GF (1997). Cocaine-but not food-seeking behavior is reinstated by stress after extinction. Psychopharmacology.

[CR2] Aklin WM, Tull MT, Kahler CW, Lejuez C (2009). Risk-taking propensity changes throughout the course of residential substance abuse treatment. Personal Individ Differ.

[CR3] Andrucci GL, Archer RP, Pancoast DL, Gordon RA (1989). The relationship of MMPI and sensation seeking scales to adolescent drug use. J Pers Assess.

[CR4] Bardo MT, Donohew RL, Harrington NG (1996). Psychobiology of novelty seeking and drug seeking behavior. Behav Brain Res.

[CR5] Billieux J, Van der Linden M, Ceschi G (2007). Which dimensions of impulsivity are related to cigarette craving?. Addict Behav.

[CR6] Bornovalova MA, Cashman-Rolls A, O'Donnell JM, Ettinger K, Richards JB, deWit H, Lejuez CW (2009). Risk taking differences on a behavioral task as a function of potential reward/loss magnitude and individual differences in impulsivity and sensation seeking. Pharmacol Biochem Behav.

[CR7] Cain ME, Saucier DA, Bardo MT (2005). Novelty seeking and drug use: contribution of an animal model. Exp Clin Psychopharmacol.

[CR8] Cain ME, Dotson WF, Bardo MT (2006). Individual differences in the effect of novel environmental stimuli prior to amphetamine self-administration in rats (Rattus norvegicus). Exp Clin Psychopharmacol.

[CR9] Cain ME, Denehy ED, Bardo MT (2008). Individual differences in amphetamine self-administration: the role of the central nucleus of the amygdala. Neuropsychopharmacology.

[CR10] Carroll ME, Lac ST (1997). Acquisition of iv amphetamine and cocaine self-administration in rats as a function of dose. Psychopharmacology.

[CR11] Carroll KM, Power ME, Bryant K, Rounsaville BJ (1993). One-year follow-up status of treatment-seeking cocaine abusers. Psychopathology and dependence severity as predictors of outcome. J Nerv Ment Dis.

[CR12] Castellani B, Rugle L (1995). A comparison of pathological gamblers to alcoholics and cocaine misusers on impulsivity, sensation seeking, and craving. Int J Addict.

[CR13] Cherpitel CJ (1999). Substance use, injury, and risk-taking dispositions in the general population. Alcohol: Clin Exp Res.

[CR14] Christensen CJ, Kohut SJ, Handler S, Silberberg A, Riley AL (2009). Demand for food and cocaine in Fischer and Lewis rats. Behav Neurosci.

[CR15] Coventry KR, Brown RIF (1993). Sensation seeking, gambling and gambling addictions. Addiction.

[CR16] Crombag HS, Bossert JM, Koya E, Shaham Y (2008). Context-induced relapse to drug seeking: a review. Philos Trans R Soc B: Biol Sci.

[CR17] Cross CP, Cyrenne DL, Brown GR (2013). Sex differences in sensation-seeking: a meta-analysis. Sci Rep.

[CR18] Dickson PE, Mittleman G (2020). Stimulus complexity and mouse strain drive escalation of operant sensation seeking within and across sessions in C57BL/6J and DBA/2J Mice. Front Behav Neurosci.

[CR19] Dickson PE, Mittleman G (2021). Strain and sex dependent effects of isolation housing relative to environmental enrichment on operant sensation seeking in mice. Sci Rep.

[CR20] Dickson PE, Roy TA, McNaughton KA, Wilcox TD, Kumar P, Chesler EJ (2019). Systems genetics of sensation seeking. Genes Brain Behav.

[CR21] Doran N, Cook J, McChargue D, Spring B (2009). Impulsivity and cigarette craving: differences across subtypes. Psychopharmacology.

[CR22] Ersche KD, Turton AJ, Pradhan S, Bullmore ET, Robbins TW (2010). Drug addiction endophenotypes: impulsive versus sensation-seeking personality traits. Biol Psychiatry.

[CR23] Flagel SB, Akil H, Robinson TE (2009). Individual differences in the attribution of incentive salience to reward-related cues: implications for addiction. Neuropharmacology.

[CR24] Flaudias V, Maurage P, Izaute M, de Chazeron I, Brousse G, Chakroun-Baggioni N (2019). Craving mediates the relation between impulsivity and alcohol consumption among university students. Am J Addict.

[CR25] Freund VA, Schulenberg JE, Maslowsky J (2021). Boredom by sensation-seeking interactions during adolescence: associations with substance use, externalizing behavior, and internalizing symptoms in a US national sample. Prev Sci.

[CR26] Galizio M, Stein F (1984). Sensation seeking and drug choice. Int J Addict.

[CR27] Gancarz AM, San George MA, Ashrafioun L, Richards JB (2011). Locomotor activity in a novel environment predicts both responding for a visual stimulus and self-administration of a low dose of methamphetamine in rats. Behav Proc.

[CR28] Gancarz AM, Ashrafioun L, San George MA, Hausknecht KA, Hawk LW, Richards JB (2012). Exploratory studies in sensory reinforcement in male rats: effects of methamphetamine. Exp Clin Psychopharmacol.

[CR29] Gancarz AM, Kausch MA, Lloyd DR, Richards JB (2012). Between-session progressive ratio performance in rats responding for cocaine and water reinforcers. Psychopharmacology.

[CR30] Gancarz AM, Robble MA, Kausch MA, Lloyd DR, Richards JB (2012). Association between locomotor response to novelty and light reinforcement: sensory reinforcement as a rodent model of sensation seeking. Behav Brain Res.

[CR31] Gancarz AM, Robble MA, Kausch MA, Lloyd DR, Richards JB (2013). Sensory reinforcement as a predictor of cocaine and water self-administration in rats. Psychopharmacology.

[CR32] Gancarz-Kausch AM, Adank DN, Dietz DM (2014). Prolonged withdrawal following cocaine self-administration increases resistance to punishment in a cocaine binge. Sci Rep.

[CR33] Gancarz AM, Wang Z-J, Schroeder GL, Damez-Werno D, Braunscheidel KM, Mueller LE, Humby MS, Caccamise A, Martin JA, Dietz KC (2015). Activin receptor signaling regulates cocaine-primed behavioral and morphological plasticity. Nat Neurosci.

[CR34] George O, Koob GF (2017). Individual differences in the neuropsychopathology of addiction. Dialogues Clin Neurosci.

[CR35] Grimm JW, Hope BT, Wise RA, Shaham Y (2001). Incubation of cocaine craving after withdrawal. Nature.

[CR36] Hall SM, Havassy BE, Wasserman DA (1991). Effects of commitment to abstinence, positive moods, stress, and coping on relapse to cocaine use. J Consult Clin Psychol.

[CR37] Homberg JR, Karel P, Verheij MM (2014). Individual differences in cocaine addiction: maladaptive behavioural traits. Addict Biol.

[CR38] Kahler CW, Spillane NS, Metrik J, Leventhal AM, Monti PM (2009). Sensation seeking as a predictor of treatment compliance and smoking cessation treatment outcomes in heavy social drinkers. Pharmacol Biochem Behav.

[CR39] Kearns DN (2019). The effect of economy type on reinforcer value. Behav Proc.

[CR40] Kearns DN, Gomez-Serrano MA, Tunstall BJ (2011). A review of preclinical research demonstrating that drug and non-drug reinforcersdifferentially affect behavior. Curr Drug Abuse Rev.

[CR41] Kelly TH, Robbins G, Martin CA, Fillmore MT, Lane SD, Harrington NG, Rush CR (2006). Individual differences in drug abuse vulnerability: d-amphetamine and sensation-seeking status. Psychopharmacology.

[CR42] Kish GB (1955). Learning when the onset of illumination is used as the reinforcing stimulus. J Comp Physiol Psychol.

[CR43] Kish GB (1966) Studies of sensory reinforcement. In: Honig W (ed) Operant behavior: areas of research and application. Appletone-Century-Crofts, New York, pp 100–159

[CR44] Kosten TA, Miserendino MJ, Haile CN, DeCaprio JL, Jatlow PI, Nestler EJ (1997). Acquisition and maintenance of intravenous cocaine self-administration in Lewis and Fischer inbred rat strains. Brain Res.

[CR45] Kravitz HM, Fawcett J, McGuire M, Kravitz GS, Whitney M (1999). Treatment attrition among alcohol-dependent men: is it related to novelty seeking personality traits?. J Clin Psychopharmacol.

[CR46] Le Moal M (2009). Drug abuse: vulnerability and transition to addiction. Pharmacopsychiatry.

[CR47] Li C, Frantz KJ (2010). Time-dependent increases in cue-induced reinstatement of sucrose seeking after sucrose self-administration in adolescence. Behav Brain Res.

[CR48] Lloyd DR, Gancarz AM, Ashrafioun L, Kausch MA, Richards JB (2012). Habituation and the reinforcing effectiveness of visual stimuli. Behav Proc.

[CR49] Lloyd DR, Kausch MA, Gancarz AM, Beyley LJ, Richards JB (2012). Effects of novelty and methamphetamine on conditioned and sensory reinforcement. Behav Brain Res.

[CR50] Logue A, Smith ME (1986). Predictors of food preferences in adult humans. Appetite.

[CR51] Lu L, Grimm JW, Hope BT, Shaham Y (2004). Incubation of cocaine craving after withdrawal: a review of preclinical data. Neuropharmacology.

[CR52] Lynne-Landsman SD, Graber JA, Nichols TR, Botvin GJ (2011). Is Sensation Seeking a Stable Trait or Does it Change Over Time?. J Youth Adolesc.

[CR53] Marra D, Warot D, Payan C, Hispard E, Dally S, Puech AJ (1998). Anhedonia and relapse in alcoholism. Psychiatry Res.

[CR54] McCabe SE, West BT, Strobbe S, Boyd CJ (2018). Persistence/recurrence of and remission from DSM-5 substance use disorders in the United States: substance-specific and substance-aggregated correlates. J Subst Abuse Treat.

[CR55] McCabe CJ, Wall TL, Gonzalez MR, Meruelo AD, Eberson-Shumate SC, Clark DB, Nooner KB, Brown SA, Tapert SF (2021). Associations of developmental imbalance between sensation seeking and premeditation in adolescence and heavy episodic drinking in emerging adulthood. Alcohol Clin Exp Res.

[CR56] McKay JR, Rutherford MJ, Alterman AI, Cacciola JS, Kaplan MR (1995). An examination of the cocaine relapse process. Drug Alcohol Depend.

[CR57] McKay JR, Alterman AI, Mulvaney FD, Koppenhaver JM (1999). Predicting proximal factors in cocaine relapse and near miss episodes: clinical and theoretical implications. Drug Alcohol Depend.

[CR58] Meszaros K, Lenzinger E, Hornik K, Füreder T, Willinger U, Fischer G, Schönbeck G, Aschauer HN (1999). The Tridimensional Personality Questionnaire as a predictor of relapse in detoxified alcohol dependents. The European Fluvoxamine in Alcoholism Study Group. Alcohol Clin Exp Res.

[CR59] Mitchell JM, Cunningham CL, Mark GP (2005). Locomotor activity predicts acquisition of self-administration behavior but not cocaine intake. Behav Neurosci.

[CR60] Müller SE, Weijers H-G, Böning J, Wiesbeck GA (2008). Personality traits predict treatment outcome in alcohol-dependent patients. Neuropsychobiology.

[CR61] Murray HW, Patkar AA, Mannelli P, DeMaria P, Desai AM, Vergare MJ (2003). Relationship of aggression, sensation seeking, and impulsivity, with severity of cocaine use. Addict Disord Treat.

[CR62] Nieva G, Valero S, Bruguera E, Andión Ó, Trasovares MV, Gual A, Casas M (2011). The alternative five-factor model of personality, nicotine dependence and relapse after treatment for smoking cessation. Addict Behav.

[CR63] Obst E, Bernhardt N, Gan G, Plawecki MH, O'Connor S, Smolka MN, Zimmermann US (2021). Sensation seeking, impulsivity, and aggression moderate sex effects on adolescent laboratory binging. Psychol Addict Behav.

[CR64] Patkar AA, Gottheil E, Berrettini WH, Hill KP, Thornton CC, Weinstein SP (2003). Relationship between platelet serotonin uptake sites and measures of impulsivity, aggression, and craving among African-American cocaine abusers. Am J Addict.

[CR65] Patkar AA, Murray HW, Mannelli P, Gottheil E, Weinstein SP, Vergare MJ (2004). Pre—treatment measures of impulsivity, aggression and sensation seeking are associated with treatment outcome for African—American cocaine—dependent patients. J Addict Dis.

[CR66] Pedersen W (1991). Mental health, sensation seeking and drug use patterns: a longitudinal study. Br J Addict.

[CR67] Piazza PV, Deminière J-M, Le Moal M, Simon H (1989). Factors that predict individual vulnerability to amphetamine self-administration. Science.

[CR68] Piazza PV, Deminiere JM, Maccari S, Mormede P, Le Moal M, Simon H (1990) Individual reactivity to novelty predicts probability of amphetamine self-administration. Behav Pharmacol 1(4):339–345 10.1097/00008877-199000140-00007 11175418

[CR69] Piazza PV, Deroche-Gamonent V, Rouge-Pont F, Le Moal M (2000). Vertical shifts in self-administration dose–response functions predict a drug-vulnerable phenotype predisposed to addiction. J Neurosci.

[CR70] Ramos-Grille I, Gomà-i-Freixanet M, Aragay N, Valero S, Vallès V (2015). Predicting treatment failure in pathological gambling: the role of personality traits. Addict Behav.

[CR71] Regan T, Thamotharan S, Hahn H, Harris B, Engler S, Schueler J, Fields SA (2020). Sensation seeking, sexual orientation, and drug abuse symptoms in a community sample of emerging adults. Behav Pharmacol.

[CR72] Reuter M, Netter P (2001). The influence of personality on nicotine craving: a hierarchical multivariate statistical prediction model. Neuropsychobiology.

[CR73] Robinet P, Rowlett J, Bardo M (1998). Individual differences in novelty-induced activity and the rewarding effects of novelty and amphetamine in rats. Behav Proc.

[CR74] Saunders BT, Yager LM, Robinson TE (2013). Preclinical studies shed light on individual variation in addiction vulnerability. Neuropsychopharmacology.

[CR75] Schenk S, Partridge B (2001). Influence of a conditioned light stimulus on cocaine self-administration in rats. Psychopharmacology.

[CR76] Sedighim S, Carrette LL, Venniro M, Shaham Y, de Guglielmo G, George O (2021). Individual differences in addiction-like behaviors and choice between cocaine versus food in Heterogeneous Stock rats. Psychopharmacology.

[CR77] Simpson DD, Joe GW, Fletcher BW, Hubbard RL, Anglin MD (1999). A national evaluation of treatment outcomes for cocaine dependence. Arch Gen Psychiatry.

[CR78] Stewart J (1960). Reinforcing effects of light as a function of intensity and reinforcement schedule. J Comp Physiol Psychol.

[CR79] Substance Abuse and Mental Health Services Administration (SAMHSA) (n.d.) 2020 National Survey of Drug Use and Health (NSDUH) releases. SAMHSA.gov. https://www.samhsa.gov/data/release/2020-national-survey-drug-use-and-health-nsduh-releases

[CR80] Tatari F, Farnia V, Momeni K, Davarinejad O, Salemi S, Soltani B, Niazi N, Alikhani M (2021). Predicting addiction potential based on sensation-seeking, psychological hardiness and assertiveness in students in western Iran: an analytical study. J Subst Use.

[CR81] Teichman M, Barnea Z, Rahav G (1989). Sensation seeking, state and trait anxiety, and depressive mood in adolescent substance users. Int J Adhes.

[CR82] Terasaki M, Imada S (1988). Sensation seeking and food preferences. Personal Individ Differ.

[CR83] Walsh SL, Donny EC, Nuzzo PA, Umbricht A, Bigelow GE (2010). Cocaine abuse versus cocaine dependence: cocaine self-administration and pharmacodynamic response in the human laboratory. Drug Alcohol Depend.

[CR84] White TL (2017). Beyond sensation seeking: a conceptual framework for individual differences in psychostimulant drug effects in healthy humans. Curr Opin Behav Sci.

[CR85] Xu X, Wu Y, Zhou S (2022). Social support and drug abstention motivation among chinese male drug addicts: a moderated mediation model of self-control and sensation-seeking. Int J Environ Res Public Health.

[CR86] Zuckerman M (1994). Behavioral expressions and biosocial bases of sensation seeking.

[CR87] Zuckerman M (2007). Sensation seeking and risky behavior.

[CR88] Zuckerman M, Eysenck S, Eysenck HJ (1978). Sensation seeking in England and America: cross-cultural, age, and sex comparisons. J Consult Clin Psychol.

[CR89] Zuckerman M, Buchsbaum MS, Murphy DL (1980). Sensation seeking and its biological correlates. Psychol Bull.

