# Supplementary material for: Operant novelty seeking predicts cue-induced reinstatement following cocaine but not water reinforcement in male rats
Source: Psychopharmacology (Berl). 2023 Aug 8;240(10):2201–15. doi: 10.1007/s00213-023-06441-4 (PMC10506955; doi:10.1007/s00213-023-06441-4)
Supplement: Supplementary file 1 — Supplementary file1 (DOCX 83 KB) [file 213_2023_6441_MOESM1_ESM.docx]

**Supplemental Figure 1.** Number of reinforcers earned during 2-h self-administration sessions (**a**) cocaine infusions, and (**b**) water deliveries.
